# Follow Up on Simple (Closed) Extraction of Fractured Maxillary Canine Teeth in Domestic Ferrets (*Mustela putorius furo*)

**DOI:** 10.3389/fvets.2021.677680

**Published:** 2021-05-13

**Authors:** Pia Kristina Primožič, Žiga Žagar, Klemen Šmalc, Joško Račnik, Tanja Švara, Ana Nemec

**Affiliations:** ^1^Dentistry and Oral Surgery Department, Small Animal Clinic, Veterinary Faculty, University of Ljubljana, Ljubljana, Slovenia; ^2^Clinic for Birds, Small Mammals and Reptiles, Institute for Poultry, Birds, Small Mammals and Reptiles, Veterinary Faculty, University of Ljubljana, Ljubljana, Slovenia; ^3^Institute of Pathology, Wild Animals, Fish and Bees, Veterinary Faculty, University of Ljubljana, Ljubljana, Slovenia

**Keywords:** canine tooth, dental fracture, dental extraction, ferret, wildlife

## Abstract

Fractured canine teeth (especially maxillary canine teeth) are common in domestic ferrets (*Mustela putorius furo*). We evaluated (detailed oral/dental and radiographic examination under general anesthesia) 18 domestic ferrets affected by 23 complicated crown fractures of periodontally healthy permanent maxillary canine teeth over a 2-year period. Average age of the ferrets at the time of diagnosis was 2.6 years. Only three teeth were discolored on clinical examination. Out of 23 teeth, 22 were radiographically evaluated. No radiographic evidence of endodontic disease was observed in 11/22 canine teeth. Inflammatory root resorption was observed in 5/22, periapical lucency in 5/22 teeth, apical widening of periodontal ligament space in 6/22 teeth, and failure of the pulp cavity to narrow in 3/22 teeth. All animals were treated by simple (closed) extraction of the affected teeth. Histological examination of eight teeth was performed. Pulp appeared histologically vital in five (3/5 showed no radiographic evidence of endodontic disease), pulpitis was diagnosed in two (both without radiographic evidence of endodontic disease) and pulp necrosis in one case (dental radiographs revealed apical widening of periodontal ligament space and failure of the pulp cavity to narrow). All extraction sites healed uneventfully by the 2-week recheck examination. Long-term follow-up revealed development of post-extraction upper lip entrapment with mandibular canine tooth in eight out of 18 ferrets, which appeared clinically irrelevant. According to clients seven out of 18 ferrets showed an improved quality of life. Simple tooth extractions are warranted in ferrets affected by complicated crown fracture of the periodontally healthy permanent maxillary canine teeth.

## Introduction

The popularity of domestic ferrets (*Mustela putorius furo*), a carnivore mammal of the *Mustelidae* family, has increased tremendously in thev recent years (1). Literature suggests oral examination should be performed as part of a routine veterinary care at least annually (2, 3).

Ferret maxillofacial anatomy and conformation is consistent with their strict carnivore diet (2). Ferrets are diphyodont with 34 permanent teeth (I 3/3; C 1/1; P 3/3; M 1/2), which erupt by the age of 50-74 days (1–4). Similarly to cats and dogs, ferrets dentition is brachyodont. Due to the similarities in dentition and skull anatomy, applying canine and feline dental guidelines to the diagnosis and treatment of dental pathology in ferrets has previously been recommended (1, 2, 5).

Reports referring to common oral and dental pathology in ferrets are rare. Among the different types of dental pathology (e.g., malocclusion, periodontal disease, canine tooth extrusion, dental abrasion, and attrition), tooth fractures are often observed and usually incidentally discovered (6). Dental fractures may occur after traumatic events such as falls, cage-biting or other forms of trauma (1, 4). Tooth fractures are mainly associated with maxillary canine teeth and commonly involve the dental pulp (1, 3). Experimentally, endodontic disease in ferrets has been shown to result in radiographically visible periapical lucencies consistent with periapical inflammation within 4 weeks (7). However, in a clinical setting only a minor proportion of fractured teeth with exposed dental pulp in ferrets show signs of endodontic disease (3). Anecdotally, monitoring has been suggested for fractured teeth in ferrets without radiographic signs of endodontic disease.

The treatment of choice for teeth affected by complicated crown fractures in dogs and cats include either endodontic therapy (8–10) or extraction due to inevitable endodontic infection as a sequel of dental pulp exposure (11, 12). Extraction of periodontally healthy canine teeth usually requires a mucogingival flap and removal of the alveolar bone (i.e., open, surgical extraction) due to long roots and possible healing complications if an oronasal communication is present (e.g., due to severe periapical disease or iatrogenic trauma) (13, 14).

To the authors' knowledge, a simple (closed) extraction of a fractured canine tooth in ferrets has not been described in the scientific literature, but may be warranted due to the friability of the mucoperiosteal tissues and clinically observed (in the authors' experience) poor tolerance of ferrets to the sutures in the oral cavity. The aims of this study were to evaluate the feasibility and outcome of simple (closed) extraction of periodontally healthy fractured permanent maxillary canine teeth in ferrets and histologically determine the condition of the dental pulp of fractured teeth with pulp exposure in association with radiographic findings.

## Materials and Methods

Medical records of client-owned domestic ferrets treated by simple (closed) extraction for a complicated crown fracture of periodontally healthy permanent maxillary canine teeth were retrospectively reviewed. Patients were initially presented to the Clinic for Birds, Small Mammals and Reptiles, Institute for Poultry, Birds, Small Mammals and Reptiles, Veterinary Faculty Ljubljana between July 2015 and May 2017 for their annual health checks, pre-vaccination examinations and diagnostic procedures to investigate different non-oral diseases as well as suspected dental problems. All ferrets with dental fractures were referred for a detailed oral and dental examination under general anesthesia to the Dentistry and Oral Surgery Department, Small Animal Clinic of the Veterinary Faculty Ljubljana. Inclusion criteria included systemically healthy animals as per clinical examination, complete blood count and blood biochemistry analysis. Client consent for diagnostics and treatment was obtained for all animals.

All animals were evaluated under general anesthesia performed following established routine protocols (15). All dental procedures were performed by a board certified veterinary dental specialist (AN). Detailed oral and dental examination included full-mouth periodontal probing and dental charting, and was performed according to the American Veterinary Dental College (AVDC) guidelines for dogs and cats and adjusted for ferrets. Clinical findings were supported by full-mouth dental radiographic examination (six or eight bisecting-angle views and two parallel views of the caudal mandibles) using the size 0 and 2 imaging plates (X-Mind Intraoral X-Ray, Satelec Acteon Group, Mérignac-Cedex, France; CR7 Vet Image X-ray Scanner and a high-resolution reusable imaging plates, iM3 Dental Limited, Stamullen, Co. Meath, Ireland) in all animals. Dental radiographs of one animal were not available for evaluation. All fractured maxillary canine teeth were evaluated for radiographic signs of endodontic disease (i.e., failure of the pulp cavity to narrow, widening of the periodontal ligament space apically, periapical lucency, integrity of the apex/inflammatory root resorption apically) (16). The findings of periodontal probing and dental charting were combined with those of the radiographic examination to obtain a diagnosis for each tooth. Thereafter, the oral cavity was rinsed with 0.12% chlorhexidine gluconate solution (CHX 0.12% oral rinse, CelsusMED GmbH, Vienna, Austria), infraorbital nerve blocks using 0.1 ml (not exceeding 1 mg/kg total dose) levobupivacaine (Chirocaine 5 mg/ml, AbbVie GmbH, Vienna, Austria) were applied as clinically indicated using an insulin syringe with a 30G needle, and periodontal treatment using an ultrasonic scaler (Cavitron Select SPS, Dentsply Sirona, Charlotte, North Carolina, USA) was performed. With an aseptic approach the affected canine tooth was luxated using a 1 mm luxator and delivered with extraction forceps. The extraction site was left to heal by second intention. All animals were discharged on the same day of the procedure with meloxicam (0.2 mg/kg PO SID 3-5 days; Metacam, Boehringer Ingelheim Vetmedica GmbH, Ingelheim, Germany). The first seven animals also received amoxicillin/clavulanic acid (12.5 - 20 mg/kg PO BID 5-7 days; Clavaseptin 62.5 mg, Vetoquinol SA, France).

All the extracted teeth were fixed in 10% buffered formalin and submitted for histopathological analysis at the Institute of Pathology, Wild Animals, Fish and Bees, Veterinary Faculty Ljubljana. After fixation, the samples were decalcified with a solution containing 12% ethylenediamine tetraacetic acid (EDTA) and 5% formic acid. The samples were shaken repeatedly at room temperature and the decalcification solution was replaced every 7 days. After decalcification, teeth were washed under running tap water for 2 and refixed in 10% buffered formalin for at least 3 days before further processing. Specimens were routinely embedded in paraffin. Four μm thick tissue sections were first deparaffinized and then stained with haematoxylin and eosin (HE). Stained sections were examined with a light microscope.

All patients were presented for a non-sedated recheck examination 2 weeks after the procedure. Phone interviews with the owners were carried out 2 months to 2 years (mean 13 months) after the procedure to acquire additional information on the post-operative recovery and long-term outcome of the procedure. A standardized set of questions included healing time, eating habits (how the animal is prehending food and how is the appetite), noticeable changes in activity levels and a subjective evaluation of quality of life.

## Results

### Population

Eighteen systemically healthy domestic ferrets (four spayed females, one intact female, six castrated, and seven intact males) were presented to the Veterinary Faculty, University of Ljubljana, Slovenia between July 2015 and May 2017 and were eligible for inclusion in the study. Their average age at the time of diagnosis was 2.6 years (3 months−5 years). Females weighed from 660 to 870 g (average 755 g) and males weighed from 830 to 2,000 g (average 1,440 g).

### Clinical and Radiographic Findings of the Fractured Teeth

**Figure 1:**
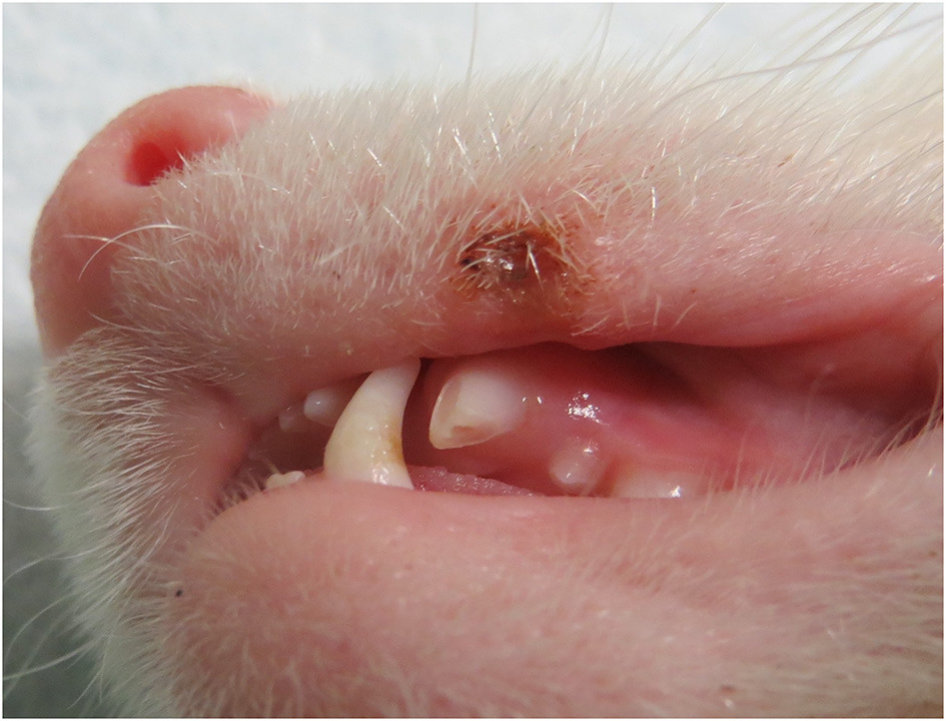
Preoperative photograph of a domestic ferret affected by a complicated crown fracture of the left maxillary canine tooth, missing the majority of the crown, and a maxillary lip lesion caused by the healthy ipsilateral mandibular canine tooth.

All dental fractures were incidentally found either by the owner or by the veterinarian. The time interval between the trauma and the diagnosis as well as the reason(s) for the dental fracture remain unknown for all of the cases. In total, 23 periodontally healthy permanent maxillary canine teeth were affected by complicated crown fractures (Figure 1). The left maxillary canine tooth was fractured in 15 out of 23 teeth (65.2%), and the right maxillary canine tooth was fractured in eight out of 23 teeth (34.8%). Five ferrets (27.8%) were affected by a fracture of both maxillary canine teeth.

Out of 23 teeth, three (13.0%) were discolored (intrinsic discoloration) on clinical examination. Tooth extrusion was observed in one case (4.3%). Upper lip lesion caused by ipsilateral mandibular canine tooth lip entrapment was found in one ferret prior to the procedure, where the fractured tooth was missing two thirds of the crown (Figure 1).

Dental radiographs of one patient were not available for evaluation. Radiographic signs of endodontic disease (16) were observed in 11 out of 22 (50.0%) fractured teeth with exposed dental pulp. In six of these 11 teeth (27.3%) an increased width of periodontal ligament space apically was found. Additionally, periapical lucency and chronic inflammatory root resorption were present in five evaluated teeth (22.7%). Failure of the pulp cavity to narrow (i.e., an increased diameter of the pulp cavity of the fractured tooth compared to the contralateral intact tooth), indicating a non-vital tooth, was found in three cases (13.6%). Among these three teeth, two were also discolored on clinical examination.

### Histological Aspects of the Fractured Teeth

Despite following an established protocol for decalcification (17), we had great difficulty making good quality histopathological slides as in some tissue sections the pulp cavity was not included, and in some cases the pulp fell out during sectioning and staining, resulting in only eight out of 23 teeth (34.8%) with sufficiently preserved dental pulp allowing histopathological evaluation.

Dental pulp appeared histologically vital in 5 (62.5%) teeth, but among these, four had histological signs of external resorption at the apex, three had congestion of pulp blood vessels and in two cases an apparently increased number of lymphocytes in blood vessels was observed. Radiographic signs of inflammatory root resorption and periapical lucency were found in two out of five teeth with histologically vital pulp.

**Figure 2:**
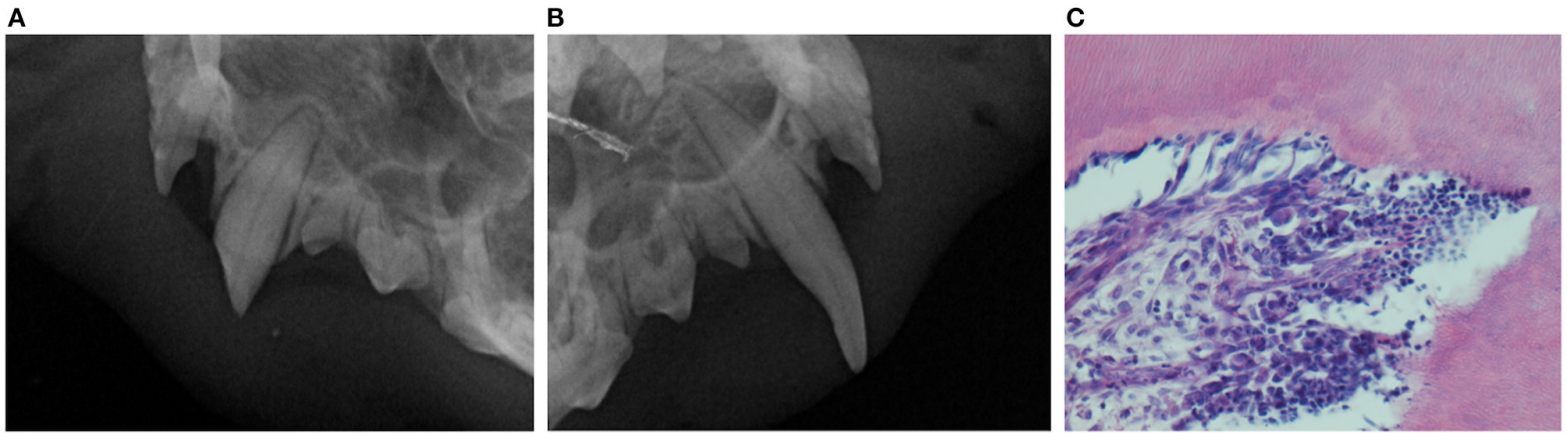
Preoperative dental radiographs of the maxillary canine teeth **(A,B)** of a representative ferret included in this study. Lateral view (bisecting angle technique) of the left maxillary canine tooth affected by a complicated crown fracture **(A)** and healthy (note only mild abrasion on the caudal aspect and the tip of the crown) contralateral maxillary canine tooth (there is slight elongation of the canine tooth and an artifact at the left side of the image not interfering with the interpretation) **(B)** presented for comparison. No radiographic signs of endodontic disease are diagnosed associated with the fractured tooth **(A)**. However, photomicrograph of the dental pulp of the fractured left maxillary canine tooth **(C)** reveals pleocellular pulpits. The pulp stroma is densely infiltrated with macrophages, lymphocytes and neutrophils.

Out of eight samples, three showed gross pathologic changes. In two of these teeth, histological examination revealed inflammation of the dental pulp. In one case a pleocellular pulpitis (Figure 2) was found and a neutrophilic pulpitis was diagnosed in the other case. No endodontic pathology was radiographically diagnosed in these two teeth (Figure 2). Only one tooth had evidence of pulp necrosis. A discoloration was observed clinically in this case, whereas radiographs revealed apical widening of periodontal ligament space and failure of the pulp cavity to narrow.

### Treatment Outcome

**Figure 3:**
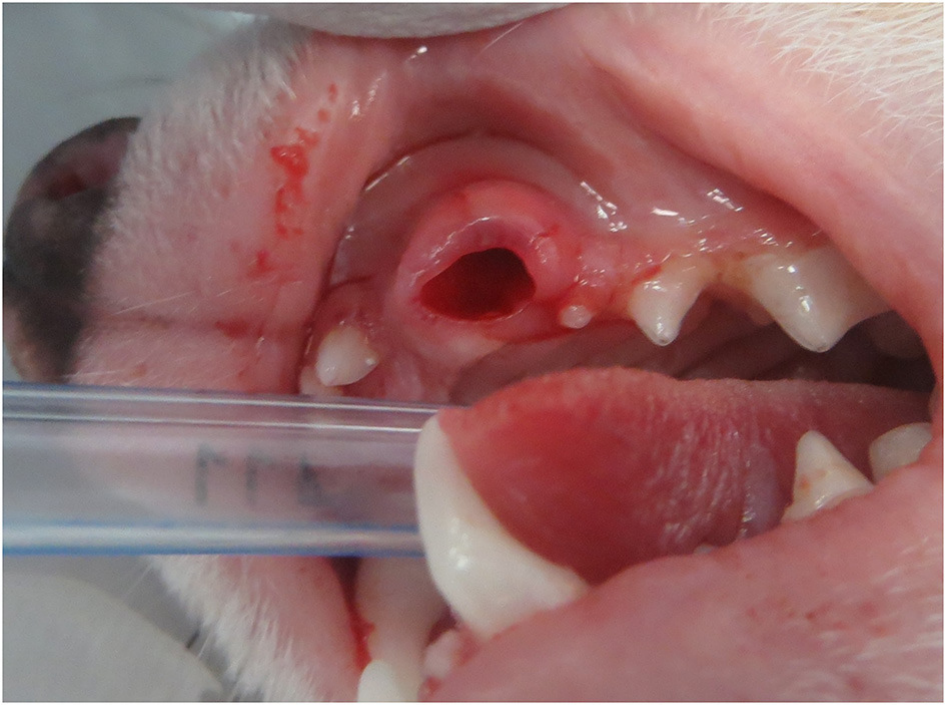
Clinical appearance of the vacated alveolus immediately after simple (closed) extraction of the periodontally healthy fractured left maxillary canine tooth.

All patients underwent simple extraction of the affected teeth without complications (Figure 3) apart from 1 animal. In this patient, an oronasal communication was clinically detected while gently exploring the vacated alveolus with a periodontal probe immediately after the extraction. This patient was presented with a discolored canine tooth and radiographic evidence of periapical lucency.

**Figure 4:**
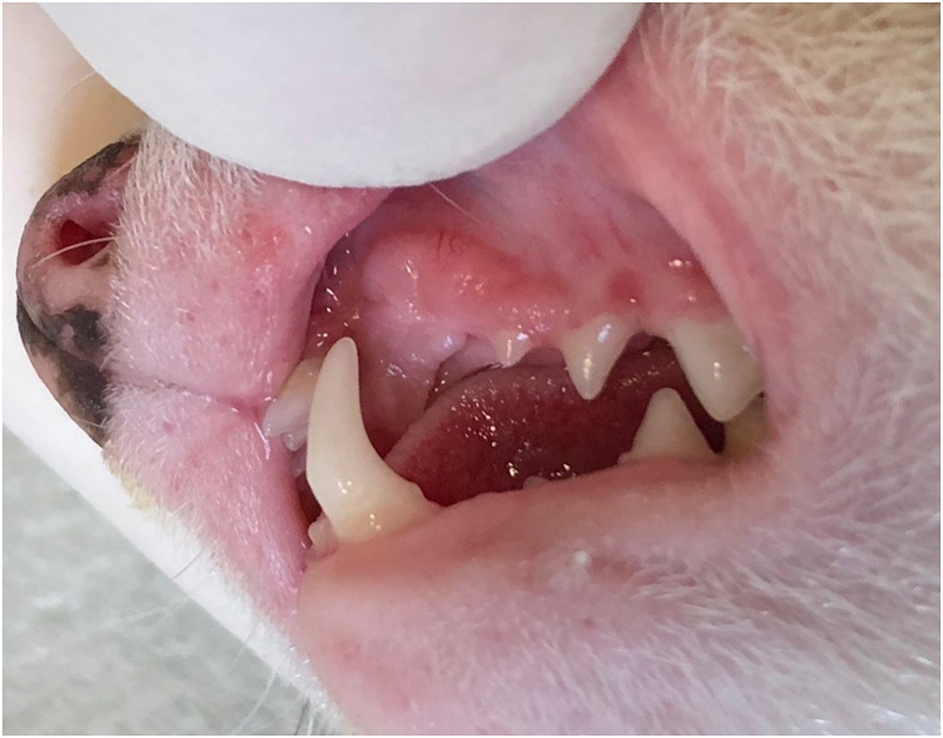
Clinical appearance of the healed extraction site at the 2-week recheck examination of the non-sedated animal.

Owners reported rapid (few days) wound healing in 14 out of 18 ferrets (77.8%). In two ferrets the owners reported persistent inflammation in the area of the extracted teeth and two other owners reported healing took more than just a few days. Minor postoperative swelling of the upper lip was reported in two ferrets (11.1%). Four (22.2%) ferrets had difficulties prehending food in the first days after the procedure, however, the signs resolved spontaneously afterwards. All ferrets healed uneventfully and completely by the recheck visit 2 weeks after the procedure (Figure 4).

As subjectively evaluated by the owners, seven (38.9%) ferrets showed an improved quality of life noted by improved appetite and food prehension, playfulness and absence of oral discomfort. In the follow-up period five (27.8%) ferrets gained weight. Post-extraction upper lip entrapment with ipsilateral mandibular canine tooth developed in eight (44.4%) ferrets. Associated upper lip lesion with minor complications not requiring further intervention was only reported in two ferrets (11.1%).

## Discussion

The presence of dental fractures in domestic ferrets is often reported, and the most commonly affected teeth are the maxillary canine teeth (1, 3), similar to reports in dogs and cats (18). Domestic ferrets may be predisposed to fracture of these teeth due to biomechanical changes of feeding as the result of captive diet, activity and environmental factors, such as abnormal chewing behavior (e.g., cage biting) (1, 4, 6, 19). They are also prone to high-rise injuries and in rare cases even exposed to human abuse, which may also lead to higher prevalence of dental fractures (20). However, the cause and timing of dental fracture in ferrets in this study remains unknown for all animals. Dental fractures are reported to be incidental findings in 35.5% in dogs (21).

Apart from missing portions of the crown, other clinical signs (e.g., discoloration) associated with fractured canine teeth in ferrets were rarely observed in this study. On the other hand, 50% of fractured teeth showed radiographic signs of endodontic disease, which is more than previously reported for fractured teeth in ferrets (3) and comparable to the reports for fractured teeth in dogs (65.6%) (11). Radiographically visible endodontic disease was experimentally induced in ferrets (dental pulp was not evaluated histologically) (7) and there is no reason to believe that naturally exposed dental pulps in ferrets remain free of inflammation and infection even without radiographic signs of endodontic disease. Indeed, none of the dental pulps of the eight teeth evaluated histologically was considered healthy, although the majority (5) were vital with only minor changes observed (e.g., blood vessel congestion and increased number of lymphocytes in blood vessels). This was an interesting observation as two of these teeth showed significant radiographic changes (i.e., inflammatory root resorption and periapical lucency). Such radiographic changes would be expected with more extensive histological changes in the dental pulp as an experimental study in dogs has shown a correlation between the histopathological condition of the pulp and the histopathological findings of periapical tissues (no dental radiographs were performed in this experiment) (22). On the other hand, in two teeth with pulpitis no endodontic pathology was diagnosed radiographically. Similar findings have been reported for dogs with fractured teeth, even for those with grossly non-vital dental pulp (11, 23). Although it is impossible to extrapolate the findings of our histological examination on all fractured teeth due to the small number of samples examined, poor correlation between radiographically detectable endodontic disease and histopathological status of the dental pulp is suggested. Unfortunately, histological examination of dental pulps of all extracted teeth in this study was not possible due to difficulties with the histopathological processing necessary to evaluate the teeth, as the contents of the pulp cavity were either not present, or the dental pulp fell out during sectioning and staining. Despite an extensive literature search, other authors who have performed histopathological studies on ferret canine teeth do not report such artifacts. We believe the cause lies in the fact that the pulp cavity is narrow and therefore difficult to get in the tissue section, and in the varying degrees of shrinkage of dentin and dental pulp during processing.

To treat endodontic disease and reduce associated discomfort in complicated crown fractures in dogs and cats, either extraction or endodontic treatment of the affected tooth is recommended. Closed extraction technique in dogs and cats is primarily warranted for small single rooted teeth, while canine teeth, especially if periodontally healthy, are recommended to be extracted with a surgical approach (13).

This report documents a successful simple (closed) extraction of periodontally healthy permanent maxillary canine teeth in ferrets, resulting in good immediate and long-term outcomes, including the case, where (presumably iatrogenic, but possibly also related to existent periapical pathology) oronasal communication was noted post-extraction. Although initially we included postoperative antibiotic therapy, we later excluded this treatment in the majority of animals to follow the standards of care for healthy dogs and cats undergoing uncomplicated dental extractions (24). No difference in healing and recovery was observed; therefore antibiotic therapy does not appear to be indicated in healthy ferrets undergoing uncomplicated simple (closed) extractions of periodontally healthy fractured permanent canine teeth.

In the majority of cases, owners perceived the procedure to have minimal effect on their animal's function in the immediate postoperative period and all ferrets completely recovered within 2 weeks. In the longer term, some owners subjectively also observed improved quality of life in their animals despite the relatively common (44.4%) post-extraction upper lip entrapment with ipsilateral mandibular canine tooth. Maxillary lip entrapment is considered common in cats following extraction of a maxillary canine tooth, especially if alveolectomy of expanded buccal bone is performed (13). None of the ferrets in this study had buccal bone expansion and one of the ferrets presented with a lesion on the maxillary lip due to lip entrapment even before canine tooth removal. This suggests that the height of the maxillary canine tooth rather than the mere presence/absence of the maxillary canine tooth and/or buccal bone shape may be important in prevention of the mandibular canine tooth touching the ipsilateral maxillary lip. It has been reported before that maxillary canine teeth deflect the upper lips away from the line of occlusion (1), hence regardless of the extraction technique used, upper lip entrapment may occur postoperatively.

Despite the relatively common occurrence of the lip entrapment in the ferrets included in this study, development of the associated upper lip lesions was only rarely (11.1%) observed. To minimize the risk of postoperative lip irritation due to self-induced trauma after maxillary canine tooth extraction, a careful odontoplasty without exposing the dental pulp of the ipsilateral mandibular canine tooth may be performed as suggested for cats (13).

In conclusion, the results of this study indicate that treatment of periodontally healthy permanent canine teeth affected by complicated fractures in ferrets is warranted, regardless of absence of radiographic signs of endodontic disease. However, further studies elucidating spontaneous endodontic disease development and progression in ferrets are needed. This study also confirms that simple (closed) extraction of periodontally healthy permanent maxillary canine teeth in ferrets is feasible and results in a rapid return to function with minimal long-term complications.

## Data Availability Statement

The original contributions presented in the study are included in the article/supplementary material, further inquiries can be directed to the corresponding author/s.

## Author Contributions

PP, ŽŽ, KŠ, JR, TŠ, and AN wrote sections of the manuscript. All authors contributed to manuscript revision, read, and approved the submitted version.

## Conflict of Interest

The authors declare that the research was conducted in the absence of any commercial or financial relationships that could be construed as a potential conflict of interest.

## References

[1] Eroshin VV Reiter AM Rosenthal K Fordham M Latney L Brown S. Oral examinations results in rescued ferrets: clinical findings. J Vet Dentistry. (2011) 28:8–15. 10.1177/089875641102800102 21696122

[2] Johnson-Delaney CA. Diagnosis and treatment of dental diseases in ferrets. J Exotic Pet Med. (2008) 17:132–7. 10.1053/j.jepm.2008.03.011

[3] Nemec A Zadravec M Racnik J. Oral and dental diseases in a population of domestic ferrets (Mustela putorius furo). J Small Animal Practice. (2016) 57:553–60. 10.1111/jsap.12546 27604062

[4] Milella L. Oral and dental conditions in ferrets. Companion Animal. (2006) 11:1–3. 10.1111/j.2044-3862.2006.tb00051.x

[5] Crossley DA. Dental disease in ferrets. In: Quesenberry K Carpenter JW, eds. Ferrets, Rabbits, and Rodents 2nd Edition: Clinical Medicine and Surgery. Philadelphia, PA: Saunders (2003). p. 374.

[6] Hoefer HL Fox JG Bell JA. Ferrets: gastrointestinal diseases. In: Quesenberry K Carpenter JW, eds. Ferrets, Rabbits, and Rodents 3rd Edition: Clinical Medicine and Surgery. Saint Louis, MO: Saunders (2012). p. 27-45. 10.1016/B978-1-4160-6621-7.00003-8

[7] Fouad AF Walton RE Rittman BR. Induced periapical lesions in ferret canines: histologic and radiographic evaluation. Dental Traumatol. (1992) 8:56–62. 10.1111/j.1600-9657.1992.tb00229.x 1521506

[8] Kuntsi-Vaattovaara H Verstraete FJ Kass PH. Results of root canal treatment in dogs: 127 cases (1995-2000). J Am Vet Med Assoc. (2002) 6:775–80. 10.2460/javma.2002.220.775 11924577

[9] Luotonen N Kuntsi-Vaattovaara H Sarkiala-Kessel E Junnila JJT Laitinen-Vapaavuori O Verstraete FJM. Vital pulp therapy in dogs: 190 cases (2001-2011). J Am Vet Med Assoc. (2014) 4:449–59. 10.2460/javma.244.4.449 24479460

[10] Thorne S Johnston N Adams VJ. Successful use of MTA Fillapex as a sealant for feline root canal therapy of 50 canines in 37 cats. J Vet Dentistry. (2020) 2:77–87. 10.1177/0898756420948140 32856554

[11] Srečnik Š Zdovc I Javoršek U Pirš T Pavlica Z Nemec A. Microbiological aspects of naturally occurring primary endodontic infections in dogs. J Vet Dentistry. (2019) 2:124–8. 10.1177/0898756419873639 31542989

[12] Rodrigues MX Nemec A Fiani N Bicalho RC Peralta S. Endodontic microbiome of fractured non-vital teeth in dogs determined by 16S rRNA gene sequencing. Front Vet Sci. (2019) 6:348. 10.3389/fvets.2019.00348 31649943 PMC6794715

[13] Lommer M. Special considerations in feline exodontics. In: Verstraete FJ Lommer M Arzi B, eds. Oral and Maxillofacial Surgery in Dogs and Cats. 2nd Edition. Saint Louis, MO: Elsevier (2020). p. 160–72. 10.1016/B978-0-7020-7675-6.00026-7

[14] Tsugawa AJ Lommer MJ Verstraete FJ. Extraction of canine teeth in dogs. In: Verstraete FJ Lommer M Arzi B eds. Oral and Maxillofacial Surgery in Dogs and Cats. 2nd ed. St. Louis, MO: Elsevier (2020). p.142–50. 10.1016/B978-0-7020-7675-6.00024-3

[15] Johnson-Delaney CA. Ferrets: anaesthesia and analgesia. In: Keeble E Meredith A, eds. BSAVA Manual of Rodents and Ferrets. Gloucester, UK: British Small Animal Veterinary Association (2009). p. 245–253. 10.22233/9781905319565.22

[16] Fiani N Arzi B. Diagnostic imaging in veterinary dental practice. J Am Vet Med Assoc. (2010) 1:41–3. 10.2460/javma.236.1.41 20043796

[17] Nemec A Pavlica Z Stiblar-Martincic D Petelin M Erzen D Crossley D. Histological evaluation of the pulp in teeth from dogs with naturally occurring periodontal disease. J Vet Dentistry. (2007) 4:212–23. 10.1177/089875640702400402 18309854

[18] Soukup JW Hetzel S Paul A. Classification and epidemiology of traumatic dentoalveolar injuries in dogs and cats: 959 injuries in 660 patient visits (2004-2012). J Vet Dentistry. (2015) 1:6–14. 10.1177/089875641503200101 26197685

[19] O'Regan HJ Kitchener AC. The effects of captivity on the morphology of captive, domesticated and feral mammals. Mammal Rev. (2005) 35:215–30. 10.1111/j.1365-2907.2005.00070.x

[20] Church B. Ferret dentition and pathology. In: Lewington JH, ed. Ferret Husbandry, Medicine and Surgery. Edinburgh, UK: Saunders (2007). p. 467–85. 10.1016/B978-0-7020-2827-4.50027-9

[21] Capik I Ledecky V Sevcik A. Tooth fracture evaluation and endodontic treatment in dogs. Acta Vet Brno. (2000) 69:115–22. 10.2754/avb200069020115

[22] Kovacević M Tamarut T Jonjić N Braut A Kovacević M. The transition from pulpitis to periapical periodontitis in dogs' teeth. Australian Endodontic J. (2008) 1:12–8. 10.1111/j.1747-4477.2008.00120.x 18352898

[23] Hale FA. Localized intrinsic staining of teeth due to pulpitis and pulp necrosis in dogs. J Vet Dentistry. (2001) 1:14–20. 10.1177/089875640101800102 11968908

[24] Sarkiala EM. Use of antibiotics and antiseptics. In: Verstraete FJ Lommer M Arzi B, eds. Oral and Maxillofacial Surgery in Dogs and Cats. 2nd ed. St. Louis, MO: Elsevier (2020). p. 160–72.

